# Evaluating Real World Health System Resource Utilization and Costs for a Risk-Based Breast Cancer Screening Approach in the Canadian PERSPECTIVE Integration and Implementation Project

**DOI:** 10.3390/cancers16183189

**Published:** 2024-09-18

**Authors:** Soo-Jin Seung, Nicole Mittmann, Zharmaine Ante, Ning Liu, Kristina M. Blackmore, Emilie S. Richard, Anisia Wong, Meghan J. Walker, Craig C. Earle, Jacques Simard, Anna M. Chiarelli

**Affiliations:** 1 HOPE Research Centre, Sunnybrook Research Institute, 2075 Bayview Avenue, Toronto, ON M4N 3M5, Canada; e25richa@uwaterloo.ca (E.S.R.); anisia.wong@sri.utoronto.ca (A.W.); 2 Sunnybrook Research Institute, Sunnybrook Health Sciences Centre, 2075 Bayview Avenue, Toronto, ON M4N 3M5, Canada; nicole.mittmann@cda-amc.ca; 3 Department of Pharmacology & Toxicology, University of Toronto, 1 King’s College Circle, Toronto, ON M5S 1A8, Canada; 4 ICES Central, 2075 Bayview Avenue, Toronto, ON M4N 3M5, Canada; zharmaine.ante@ices.on.ca (Z.A.); ning.liu@ices.on.ca (N.L.); craig.earle@ices.on.ca (C.C.E.); 5 Ontario Health, 525 University Avenue, 5th Floor, Toronto, ON M5G 2L3, Canada; kristina.blackmore@ontariohealth.ca (K.M.B.); meghan.walker@ontariohealth.ca (M.J.W.); anna.chiarelli@ontariohealth.ca (A.M.C.); 6 Dalla Lana School of Public Health, University of Toronto, Toronto, ON M5S 1A1, Canada; 7 Research Center, University Hospital Center (CHU)-Laval University, Québec City, QC G1V 4G2, Canada; jacques.simard@crchudequebec.ulaval.ca; 8 Department of Molecular Medicine, Faculty of Medicine, Université Laval, Quebec City, QC G1V 4G2, Canada

**Keywords:** breast cancer, breast cancer screening, risk stratification, economic evaluation, healthcare resource utilization

## Abstract

**Simple Summary:**

There is a current gap in understanding the costs and healthcare resources used related to breast cancer risk assessment and screening. Risk-stratified breast screening overcomes various limitations of age-based screening, and participants are classified based on personalized differences in breast cancer risk. The overall goal of the PERSPECTIVE I&I project was to inform the implementation of more effective strategies of risk assessment for risk-stratified screening and earlier detection of breast cancer. This study appears to be the first to examine the healthcare utilization and costs stratified by the three breast cancer risk levels, determining the economic burden and resource needs linked with different risk categories. This study demonstrated that despite higher screening-related costs for high-risk individuals, overall healthcare costs were comparable across risk categories.

**Abstract:**

Background: A prospective cohort study was undertaken within the PERSPECTIVE I&I project to evaluate healthcare resource utilization and costs associated with breast cancer risk assessment and screening and overall costs stratified by risk level, in Ontario, Canada. Methods: From July 2019 to December 2022, 1997 females aged 50 to 70 years consented to risk assessment and received their breast cancer risk level and personalized screening action plan in Ontario. The mean costs for risk-stratified screening-related activities included risk assessment, screening and diagnostic costs. The GETCOST macro from the Institute of Clinical Evaluative Sciences (ICES) assessed the mean overall healthcare system costs. Results: For the 1997 participants, 83.3%, 14.4% and 2.3% were estimated to be average, higher than average, and high risk, respectively (median age (IQR): 60 [56–64] years). Stratification into the three risk levels was determined using the validated multifactorial CanRisk prediction tool that includes family history information, a polygenic risk score (PRS), breast density and established lifestyle/hormonal risk factors. The mean number of genetic counseling visits, mammograms and MRIs per individual increased with risk level. High-risk participants incurred the highest overall mean risk-stratified screening-related costs in 2022 CAD (±SD) at CAD 905 (±269) followed by CAD 580 (±192) and CAD 521 (±163) for higher-than-average and average-risk participants, respectively. Among the breast screening-related costs, the greatest cost burden across all risk groups was the risk assessment cost, followed by total diagnostic and screening costs. The mean overall healthcare cost per participant (±SD) was the highest for the average risk participants with CAD 6311 (±19,641), followed by higher than average risk with CAD 5391 (±8325) and high risk with CAD 5169 (±7676). Conclusion: Although high-risk participants incurred the highest risk-stratified screening-related costs, their costs for overall healthcare utilization costs were similar to other risk levels. Our study underscored the importance of integrating risk stratification as part of the screening pathway to support breast cancer detection at an earlier and more treatable stage, thereby reducing costs and the overall burden on the healthcare system.

## 1. Introduction

Breast cancer is a major cause of morbidity and mortality in Canada and is the second leading cause of cancer-related death among Canadian females [1]. In 2022, the most commonly diagnosed cancer in Ontario was estimated to be female breast cancer, including 12,531 cases or 13.1% of all new cancer cases [2]. Canadian breast cancer screening guidelines recommend an age-based approach, advising individuals aged 50–74 to screen with mammography every two to three years [3,4]. In the United States, breast cancer screening guidelines have been recently updated to recommend biennial screening mammograms in women aged 40–74 [5]. The Ontario Breast Screening Program (OBSP) screens high-risk people aged 30 to 69 with mammography and breast magnetic resonance imaging (MRI) annually [6]. High-risk participants are those with a lifetime risk of ≥25%, high-risk germline mutation, or a history of thoracic radiation treatment between ages 10 and 30—representing fewer than 1 in 100 women in Ontario [7,8].

The age-based approach to screening according to guidelines may fail to account for population heterogeneity in breast cancer risk, with potential over- or under-screening according to their actual risk. Breast cancer is a heterogeneous disease, and evaluating risk involves examining various modifiable and non-modifiable risk factors. Non-modifiable breast cancer risk factors include family history (of breast or ovarian cancer), female sex, older age, genetic mutations (e.g., *BRCA1* or *BRCA2*), common low-penetrance single nucleotide polymorphism (SNP) genetic variants, race/ethnicity and density of breast tissue [9,10]. Modifiable breast cancer risk factors include the use of hormonal replacement therapy, being overweight/obese and alcohol intake [9,10]. Each genetic variant may confer a small risk; however, the effects of common genetic variants can be combined into polygenic risk scores (PRS) that enable breast cancer risk stratification to be applied to the general population [11]. Previous studies by Pashayan and colleagues used simulation models—such as the life-table model—to demonstrate that risk-stratified screening for breast cancer can improve cost-effectiveness and benefit-to-harm ratio compared to age-based breast cancer screening [11,12,13]. Our recent study reinforces the importance of a comprehensive, risk-based approach to breast cancer screening recommendations and is tailored to an individual’s risk for breast cancer estimated via a multifactorial risk assessment [14].

Several international studies have investigated the acceptability, efficiency, feasibility, health system readiness and ethical, psychological, socio-economic and legal issues of risk-based assessment and screening in a cancer setting [15,16,17,18,19,20,21]. Risk-based screening strategies have been proposed to tailor screening approaches based on individual risk profiles, with high-risk participants often requiring more frequent screening with advanced imaging modalities, potentially improving early detection and survival outcomes [18,19,20]. Additionally, economic evaluations have assessed the cost-effectiveness of risk stratification strategies in breast cancer management, highlighting the value of risk-based approaches in optimizing resource allocation [21,22,23]. Previous studies investigating cost-effectiveness have revealed that less screening in low-risk participants and more frequent mammography screening in high-risk participants was more cost-effective compared to no screening and age-based screening [13,21,22]. The resources saved with the reduced cost of risk-based screening can be allocated towards enhancing screening for participants at high risk of fast-growing disease, who are frequently identified between screenings, and improving overall adherence. Moreover, two previous Ontario-based population cost studies by Mittmann and colleagues investigated the total health resource costs, screening costs and diagnostic costs associated with participants at average risk and higher than average risk for breast cancer [23,24].

Despite important research into the cost-effectiveness of risk-stratified screening, there remains a notable gap in understanding how healthcare utilization and costs are impacted by population breast cancer risk assessment, particularly within a Canadian context. The PERSPECTIVE I&I project aims to address this gap by leveraging real-world evidence to investigate healthcare utilization and costs stratified by breast cancer risk level for screening in Ontario, Canada [25]. The findings will provide valuable insights into the economic impact of risk-based approaches to breast cancer care, informing the development of more effective and efficient strategies for risk assessment, screening, diagnosis, treatment and survivorship care.

## 2. Materials and Methods

### 2.1. Study Design and Data Sources

This was a prospective cohort study that included asymptomatic females aged 50 to 70 years who participated in the PERSPECTIVE I&I project undergoing risk assessment for breast cancer and who had a valid health card number (i.e., Ontario Health Insurance Plan (OHIP) card) between 1 July 2019 and 31 December 2022. The index date was defined as the study start date, which is when the entry questionnaire was completed. The recruitment of participants in the PERSPECTIVE I&I cohort involving risk-stratified breast cancer screening in a Canadian population setting has been previously published [14]. The start of the study was marked by the completion of the entry questionnaire by the participants (there were a total of three questionnaires during the study period). The end of the study and cost data collection period was defined by death, breast cancer diagnosis, end of OHIP eligibility or end of follow-up (i.e., 31 December 2022), whichever was earliest. The original patient data within the PERSPECTIVE I&I project was extracted using questionnaires, breast density evaluation, saliva samples and PRS as previously reported [14].

**Figure 1:**
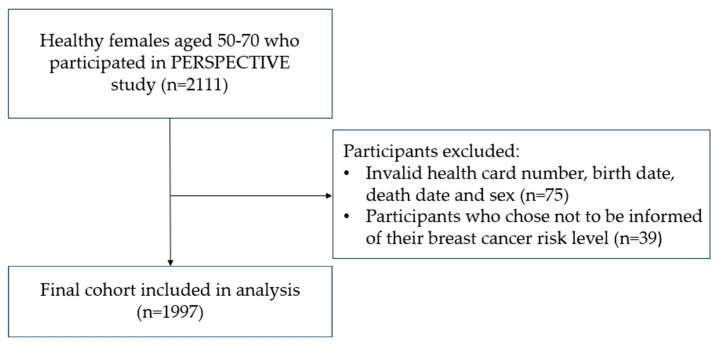
Study cohort diagram.

Exclusion criteria included females with an invalid health card number, birth date, death date (if death occurred before the index date) and sex, and participants who chose not to be informed of their breast cancer risk level. As seen in Figure 1, the final cohort in the analysis was 1997.

Participants in the PERSPECTIVE cohort were linked to real-world, population-level data from Ontario provincial databases at ICES, which collects data on public health care coverage and other population-level health information. To determine the trajectory of care over time of a cohort undergoing risk assessment, health information on each individual was linked to applicable datasets.

Data sources were accessed through ICES, which collects population-level health information in Ontario. The datasets used included the following: Canadian Institute for Health Information (CIHI) Continuing Care Reporting System (CCRS), CIHI Discharge Abstract Database (DAD), Ontario Home Care Database (HCD), CIHI National Ambulatory Care Reporting System (NACRS), CIHI National Rehabilitation Reporting System (NRS), Ontario Breast Screening Program (OBSP), Ontario Cancer Registry (OCR), Ontario Drug Benefit (ODB) Formulary, Ontario Mental Health Reporting System (OMHRS), New Drug Funding Program (NDFP), OHIP billings, Registered Persons Database (RPDB) and CIHI Same Day Surgery (SDS). These datasets included health information regarding demographics/population, diagnosis, health services, care providers and facilities. A complete description of all the databases utilized in this study is found in Appendix A and has also been described in the previous literature [26].

### 2.2. Breast Cancer Risk Estimation

Baseline characteristics that were assessed at the date of the entry questionnaire include age, ethnicity, breast density, rurality, neighborhood income quintile, Charlson comorbidity group, employment status and education status. To categorize the breast density of participants, mammogram reports were obtained from the electronic hospital records of six participating OBSP sites [9]. The Breast Imaging Reporting Data System (BI-RADS^®^) was used to classify breast density as follows: A (almost entirely fatty), B (scattered density), C (heterogeneously dense) or D (extremely dense) [27,28]. Where mammographic density was recorded on the report as “≥75%”, breast density was classified as BI-RADS^®^ category D. On 26.7% of mammogram reports, breast density was reported only as “<75%”. To avoid excluding these participants from the analysis [14], breast density was categorized as BI-RADS^®^ A/B/C. For all other participants, the breast density included in the mammogram report was abstracted. It should be noted that the Charlson Comorbidity Index (CCI) is a measure of classifying comorbid conditions impacting mortality in longitudinal studies and was used to assess comorbidity in this study [29,30].

To calculate the PRS for breast cancer for study participants, an accredited molecular lab at the Princess Margaret Cancer Center extracted DNA from saliva samples provided by the study participants to perform a clinical-grade breast cancer genetic risk SNP test [9]. The combined effect of 295 SNPs determined using Next Generation Sequencing in a prior PERSPECTIVE study was summarized in an algorithm and used to calculate the PRS [31,32]. The Breast and Ovarian Analysis of Disease Incidence and Carrier Estimation Algorithm (BOADICEA)-specific parameters for this PRS have been published elsewhere [32]. The PRS accounts for the multiplicative effects of common low-penetrance SNPs associated with breast cancer risk [16,33].

To estimate participants’ risk, the standardized PRS (beta), breast density, and additional risk factors were entered into the CanRisk web tool, which is a web-based software released in November 2019 based on the BOADICEA model. The CanRisk tool generates a personalized cancer risk assessment by integrating various breast cancer risk factors (e.g., breast density, family history of cancer, genetic variants and lifestyle/hormonal/clinical features) and applying risk prediction models [9,32,33,34]. In our study, the effect of rare pathogenetic variants in moderate and high-risk susceptibility genes was not tested and those with previous genetic counseling or testing were excluded [14]. The CanRisk tool estimated age-specific 10-year breast cancer risk stratified into three risk categories according to risk groupings used in the OBSP and some other Canadian screening programs. The three breast cancer risk categories align with the remaining lifetime (RLT) risk for people aged 30–80 years and include the following: average risk (<15% RLT), higher than average risk (15% to <25% RLT) and high risk (≥25% RLT) [12,14].

### 2.3. Statistical and Costing Analysis

Statistical analyses were conducted at ICES and performed using SAS Enterprise Guide 8.3. Overall total and mean cost per participant were reported in 2022 CAD. The total cost for screening-related activities consisted of risk assessment, total screening and total diagnostic costs. The risk assessment cost was based on PERSPECTIVE I&I cohort-linked data and was calculated by adding the cost of the PRS test, risk assessment events and genetic counseling events. The breast cancer screening costing utilized the GETBCSCOST methodology based on OHIP and OBSP data. The total screening cost includes OHIP screening mammogram, OBSP administrative data, OBSP facility and non-OBSP overhead screening costs [24]. The total diagnostic cost was calculated using the costs of diagnostic mammograms, ultrasound, magnetic resonance imaging (MRI), biopsy, diagnostic genetic, OBSP diagnostic follow-up and overhead diagnostic costs.

A macro-based costing methodology called GETCOST was used to assess the overall healthcare system costs, which is accessible through ICES. The GETCOST macro allocates healthcare costs to individual participants by determining each individual’s encounters (i.e., utilization) with the healthcare system and then applying the associated unit costs/prices to services used during these encounters [35]. The various databases and cost components used in the calculation of health system costs using the GETCOST macro include OHIP, NACRS, DAD, ODB, NRS, CCRS, HCD and OMHRS, which are further described in Appendix A. The validity of GETCOST methodology has been described in previous studies [24,26,35,36,37]. The mean cost per individual was calculated by dividing the cohort’s total cost by the number of participants alive and eligible at the start of a certain year. Moreover, the denominator when calculating the mean cost per individual included participants who had non-zero costs and used the resources at any time during follow-up.

## 3. Results

### 3.1. Socio–Demographic and Health Characteristics

Between 1 July 2019 and 30 December 2022, 2111 participants underwent risk assessment for breast cancer as part of the PERSPECTIVE I&I project in Ontario, Canada. After excluding participants without a valid health card number, birth date, death date and sex, as well as participants who were not informed of their breast cancer risk, 1997 participants were included in the final analysis. Table 1 summarizes the baseline characteristics of the participants included in the analysis, stratified by their breast cancer risk levels: average risk, higher than average risk and high risk. Notable findings include lower mean and median ages of participants in the higher-risk groups, with a higher proportion of participants 50 to 60 years of age in the higher-than-average and high-risk groups. Breast density, measured by BI-RADS, revealed that higher-risk groups included a higher proportion of participants with extremely dense breast tissue. The majority of the cohort captured was of Caucasian background and lived in urban areas. More than half were employed. Socioeconomic characteristics were not significantly different between risk level groups, with the majority of participants in the cohort within the middle- and upper-income quintiles and having obtained a bachelor’s degree or higher. The high-risk group was more likely to have a first or second-degree relative with breast, ovarian, pancreatic or prostate cancer compared to the higher-than-average and average-risk groups. In terms of other comorbidities, the majority of participants in the cohort had either a 0 Charlson Comorbidity Score or indicated no prior healthcare utilization. Almost all participants reached the end of the study period, with the exception of 18 being censored due to a new breast cancer being diagnosed. All 18 new breast cancers were early stage.

### 3.2. Breast Cancer Risk Assessment and Screening Frequency

Table 2 shows the breast cancer risk assessment and screening frequency for all participants and stratified by risk level. Although all participants had a PRS test and risk assessment, those at high risk were more likely to have genetic counseling (100%) compared to those at average risk (7%) or higher than average risk (17%). Overall, 90% of participants had a screening mammogram and 10% had a diagnostic mammogram, with the greatest proportions in those at high risk. About 36% of high-risk participants had a breast MRI/ultrasound compared to 8% for higher than average and 6.5% for average risk. However, the mean number (±SD) that had breast MRI/ultrasound events was 1.40 (±0.80), 1.43 (±0.79) and 1.44 (±0.73) for average risk, higher than average risk and high-risk participants, respectively, and did not differ by risk group.

### 3.3. Costs

#### 3.3.1. Risk-Stratified Screening-Related Costs

Table 3 shows the risk assessment and screening-related costs for all participants and stratified by risk level. The mean screening-related cost per participant (with SD) increased as the risk level increased. The high-risk participants had the highest overall mean cost of CAD 905 (±269), mean risk assessment cost of CAD 612 (±15) and total screening cost of CAD 143 (±55) compared to average-risk and higher-than-average risk participants. The major cost burden across each risk group was the risk assessment cost, with mean cost (±SD) values of CAD 377 (±64), CAD 402 (±93) and CAD 612 (±15) for average risk, higher than average risk and high risk, respectively. Following the risk assessment costs, the next major cost drivers were total diagnostic costs and screening costs, with mean costs (±SD) of CAD 328 (±191) and CAD 120 (±43), respectively, across all risk groups.

#### 3.3.2. Healthcare Utilization Resource Costs

The overall healthcare costs from the study initiation to the end of follow-up were investigated using the GETCOST macro methodology and depicted in Table 4. The mean overall healthcare cost per participant (±SD) was the highest for average-risk participants with CAD 6311 (±19,641), followed by higher-than-average risk with CAD 5391 (±8325) and high risk with CAD 5169 (±7676). Compared to the other risk groups, the average risk group had higher mean costs per participant for inpatient costs, non-physician costs, NDFP drug costs, oral drug costs, outpatient costs and same-day surgery costs.

## 4. Discussion

Our study was a follow-up to the PERSPECTIVE I&I prospective cohort study that provided an opportunity to evaluate costs and healthcare resource utilization associated with breast cancer risk assessment and screening and overall costs stratified by risk level, in Ontario, Canada. Despite the disruption to study recruitment during the COVID-19 pandemic, a total of 1997 participants aged 50 to 70 underwent risk assessment to assess their breast cancer risk level. Among them, 83.3%, 14.4% and 2.3% were categorized as average, higher than average, and high risk, respectively. The breast cancer risk assessment and screening costs were evaluated for each risk level. Of the screening-related costs, the greatest cost burden across all risk groups was the risk assessment cost, followed by total diagnostic and screening costs. However, high-risk participants had higher mean risk-stratified screening-related costs compared to the average-risk and higher-than-average risk participants. By accurately identifying and targeting higher-risk individuals, the risk-stratification model reduces the need for extensive risk assessment, including genetic counseling and additional screening across other risk groups. An important observation was that the overall healthcare utilization costs were similar for all participants regardless of their risk stratification.

Several previous studies have performed economic evaluations assessing the cost-effectiveness of risk stratification strategies in breast cancer management, highlighting the value of risk-based approaches in optimizing resource allocation [13,21,22,23]. The economic analysis to optimize personalized risk-based screening using the multifactorial CanRisk web tool was an important objective of the PERSPECTIVE I&I project described in this study [25]. A recent systematic review by Khan et al. (2021) analyzing 10 modeling papers evaluated risk-based strategies against no screening and age-based screening in terms of cost-effectiveness. Their results demonstrated that less frequent screening for low-risk participants and more frequent mammography screening for high-risk participants were highly cost-effective compared to no screening and age-based screening [21]. Additionally, Khan et al. demonstrated that risk-based screening according to multiple risk factor criteria is cost-effective compared to no screening or age-based screening, with cost savings and quality-adjusted life year gains reported. Another study by Badal et al. (2023) investigated data from the FDA Mammography Quality Standards Act and Program in the United States for 2019 to 2021 [22]. The results suggested that the estimated yearly aggregate cost of screening all eligible participants was the highest for annual screening with USD 26.71B (USD 24.61–32.82B) compared to risk-based screening with USD 9.60B (USD 8.88–11.70B) in 2023 USD. The study also emphasized that reduced costs through risk-based screening enable resources to be redirected towards improving screening for participants at high risk of fast-growing disease [22]. A third study by Pashanyan et al. (2018) examined the cost-effectiveness and benefit-to-harm ratio of breast cancer screening programs in the United Kingdom (UK) compared to the “one-size-fits-all” age-based breast cancer screening [13]. A hypothetical cohort of 364,500 participants aged 50 years in the UK, with follow-up to age 85 years was investigated. From July 2016 to September 2017, risk-stratified screening for lower-risk versus higher-risk thresholds cost GBP 20,066 (USD 26,888) versus GBP 537,985 (USD 720,900) less, had 26.7% versus 71.4% fewer overdiagnoses and avoided 2.9% versus 9.6% fewer breast cancer deaths, respectively. The authors concluded that risk-based breast cancer screening, as opposed to age-based screening, can enhance cost-effectiveness, reduce overdiagnosis and maintain the benefits of the screening program [13]. Overall, these economic studies highlighted various advantages of risk-stratified screening, such as cost-effectiveness, reduced overdiagnosis and improved benefit-to-harm ratio—reinforcing the use of risk-stratified screening-related activities investigated in this study.

Despite the economic perspective, it is important to consider Canadian women’s attitudes towards and willingness to participate in breast cancer risk-stratified screening. A population-based cross-sectional survey study by Mbuya Bienge and et al. (2021) investigated 4293 women aged 30 to 69 in four Canadian provinces [38]. The results revealed that most women (63.5% to 72.8%) had favorable attitudes towards breast cancer risk-stratified screening. Most women (85.9%) accepted an increase in screening frequency if they were at high risk, but fewer (49.3%) accepted a reduction in screening frequency if they were at lower risk for breast cancer. This study suggested that the implementation of risk-stratified screening would be well-supported by Canadian women. It also strengthens the relevance of conducting economic evaluations of risk-stratified screening similar to our study.

Few cost studies regarding breast cancer screening stratified by risk have been conducted in Canada. Mittmann et al. (2023) performed an Ontario-based retrospective cohort study in 2023 examining concurrent cohorts of 644,932 participants aged 50–74 classified as higher than average risk and screened annually due to the risk factors of family/personal history, dense breasts (≥75%) or being screened biennially in the OBSP [23]. For higher-than-average risk participants aged 50 to 74, mean healthcare costs in 2018 CAD overall in the first year after screening were highest among those screened annually for family/personal history (CAD 4685) and lowest for those with density ≥ 75% (CAD 3366) compared to biennially (CAD 3767). The overall healthcare resource utilization cost for higher-than-average risk participants in our study was CAD 5267 (±8178), which is slightly higher but similar to the cost of those screened annually for family/personal history (CAD 4685) in Mittmann et al. (2023) [23]. Differences in costs could be attributable to the use of different risk stratification criteria, since Mittmann et al. (2023) [23] did not include PRS or use a multifactorial risk model (CanRisk tool) in their risk stratification. Moreover, the majority of participants (53.3%) with breast density ≥ 75% in our study (i.e., BI-RADS^®^ class D) were considered high risk rather than higher than average risk, as our study considered other risk factors in addition to breast density. Another population-level study by Mittmann et al. (2022) [24] investigated 1,546,386 asymptomatic participants aged 49–74 years in Ontario who had not undergone risk assessment for breast cancer between January 2013 and December 2019 [24]. The participants were monitored during a “screening episode”, defined as 8 months from the date of their screening mammogram until the maximum time expected to complete diagnostic procedures. When investigating the entire Ontario cohort (i.e., both OBSP and non-OBSP cohorts) of average risk participants in Ontario, the mean total screening cost per individual was CAD 103 ± CAD 8, and the mean total diagnostic cost per individual per month was CAD 219 ± CAD 165. In our study, the average risk participants had a mean total screening cost per individual of CAD 116 ± CAD 38 and the mean total diagnostic cost per individual per year was CAD 318 ± CAD 185. In both studies, the mean total screening costs per individual were similar; however, the diagnostic costs were higher as genetic assessment costs were included in the PERSPECTIVE I&I project.

While higher-risk participants may incur greater screening costs, reducing these costs might not be feasible as recent guidelines like the US Preventive Services Task Force include a younger population [8]. The shift towards starting screening at younger ages may be in response to the rising incidence of breast cancer among women younger than 50 years of age [39]. A recent global study highlighted that those women with a family history of breast cancer who began biennial screening at 40 instead of 50 years old experienced a 36% increase in life-years gained and a 20% reduction in breast cancer death [40]. Furthermore, breast cancer screening will likely continue to increase as the Canadian population ages, and most breast cancer cases (83%) are diagnosed in women aged over 50 years [41]. Rather than reducing current screening-related costs, the future goal is implementing risk assessment as part of risk-stratified screening to enhance breast cancer detection at an earlier and less costly stage, reducing the overall burden on the healthcare system [42,43].

This present study has various strengths to highlight. These include the large prospective cohort and the ability to collect extensive participant information on sociodemographic and risk factors, and breast cancer screening costs using questionnaire data and data linked from population health resources utilization level information. Another strength was the inclusion of risk factors from a self-reported entry questionnaire, standardized PRS from a genetic test using DNA extracted from a saliva sample and breast density on mammogram reports categorized using BI-RADS^®^ analyzed using the CanRisk web tool.

There are some limitations that may influence the external validity of our findings. Firstly, when considering baseline characteristics, the results may not be generalizable to other populations. The under-representation of participants who were visible minorities and had lower educational attainment may skew results, considering the disparities in risk factors such as socioeconomic status, biological and genetic differences in tumors, differential access to health care and disease-related molecular differences across different racial and ethnic groups [44]. This under-representation aligned with the demographic information provided in the PERSPECTIVE I&I prospective cohort compared to CanPath (Ontario and Quebec; Canada) and Canada census sociodemographic characteristics [14]. Furthermore, the impact of COVID-19 on healthcare utilization and screening-related costs for participants participating from 2019 to 2022 was not assessed in our study. Other studies have demonstrated that COVID-19 has significantly decreased rates of breast cancer screening and other cancer care services in Ontario in accordance with updated safe practice guidelines during the pandemic, resulting in 20.7% fewer cancer care services during the first year of the pandemic [45,46,47,48]. Moreover, a study limitation was the relatively short period following risk assessment and the consideration of only a portion of the total lifetime costs. Given that the study concluded and the costing data collection period ended with a maximum date of 31 December 2022, some participants may have undergone fewer screenings, therefore impacting the costing data in this study. Also, 18 participants were censored due to new breast cancer being diagnosed. It was mentioned that all new cancer cases were in the early stage and could not be reported or stratified by the three risk groups due to small cell suppression. Despite these limitations, our overall findings provide useful information for implementation of risk-based screening programs. Although the high-risk group had the highest risk assessment and screening costs, their overall healthcare system costs were similar to the other two lower-risk groups.

## 5. Conclusions

In conclusion, this prospective cohort study conducted as part of the PERSPECTIVE I&I project provided valuable insights into the costs and healthcare resource utilization associated with breast cancer risk assessment and screening in Ontario, Canada. Rather than focusing on reducing screening-related costs, our study underscored the importance of integrating risk stratification as part of the screening pathway in identifying personalized risk. This could support the detection of breast cancers at an earlier and more treatable stage, thereby reducing costs and the overall burden on the healthcare system. This pioneering effort aimed to provide critical insights into the economic burden associated with risk-stratified screening-related activities, ultimately guiding the development of more targeted and efficient breast cancer screening and management strategies tailored to the Canadian context.

## Figures and Tables

**Table 1:** Socio–demographic and health characteristics of Ontario participants aged 50–70, overall and by breast cancer risk level.

Characteristic	Total *N* = 1997	Average Risk *N* = 1664	Higher than Average Risk *N* = 288	High Risk *N* = 45	*p*-Value
Age at study initiation					
Mean (SD)	60.2 (5.0)	60.6 (5.0)	58.4 (4.6)	56.9 (3.7)	<0.0001
Median (Q1–Q3)	60 (56–64)	61 (57–65)	58 (55–62)	57 (54–59)	<0.0001
Min–max	50–70	50–70	50–68	50–65	
Age group					
50–60 years	893 (44.7%)	695 (41.8%)	164 (56.9%)	34 (75.6%)	<0.0001
60–70	1104 (55.3%)	969 (58.2%)	124 (43.1%)	11 (24.4%)	
Ethnic origin					
White	1714 (86.7%)	1451 (88.1%)	234 (82.1%)	29 (64.4%)	
East Asian	90 (4.6%)	62 (3.8%)	22 (7.7%)	6 (13.3%)	
Mixed	31 (1.6%)	21 (1.3%)	* 5–9	* 1–5	
Black	30 (1.5%)	19 (1.2%)	* 3–7	* 4–8	
Southeast Asian	29 (1.5%)	22 (1.3%)	* 2–6	* 1–5	
Latin American	26 (1.3%)	20 (1.2%)	* 3–7	* 1–5	
Indigenous	24 (1.2%)	* 19–23	* 1–5	0 (0.0%)	
South Asian	22 (1.1%)	* 17–21	* 1–5	0 (0.0%)	
West Asian	* 6–10	* 4–8	* 1–5	* 1–5	
Arab	* 1–5	* 1–5	0 (0.0%)	0 (0.0%)	<0.0001
Breast Density (BI-RADS)					
Almost entirely fatty (A)/scattered fibroglandular (B), heterogeneously dense (C)	1790 (89.6%)	1556 (93.5%)	213 (74.0%)	21 (46.7%)	<0.0001
Extremely dense (D)	207 (10.4%)	108 (6.5%)	75 (26.0%)	24 (53.3%)	
Family history of any breast, ovarian, prostate or pancreatic cancer					
No family history	893 (44.7%)	793 (47.7%)	90 (31.3%)	10 (22.2%)	
First-degree relative with cancer	385 (19.3%)	307 (18.4%)	70 (24.3%)	8 (17.8%)	<0.0001
Second-degree relative with cancer	480 (24.0%)	395 (23.7%)	70 (24.3%)	15 (33.3%)	
Both first and second-degree relatives with cancer	239 (12.0%)	169 (10.2%)	58 (20.1%)	12 (26.7%)	
Rural/Small Town					
No	1867 (93.6%)	1552 (93.4%)	* 271–275	* 40–44	0.6839
Yes	128 (6.4%)	110 (6.6%)	* 13–17	* 1–5	
Neighborhood income quintile					
1	202 (10.1%)	167 (10.0%)	27 (9.4%)	8 (17.8%)	0.3522
2	338 (16.9%)	290 (17.4%)	42 (14.6%)	6 (13.3%)	
3	368 (18.4%)	311 (18.7%)	50 (17.4%)	7 (15.6%)	
4	386 (19.3%)	323 (19.4%)	52 (18.1%)	11 (24.4%)	
5	701 (35.1%)	571 (34.4%)	117 (40.6%)	13 (28.9%)	
Employment status					
Working	1091 (55.0%)	894 (54.0%)	166 (58.5%)	31 (68.9%)	0.0124
Retired	732 (36.9%)	634 (38.3%)	90 (31.7%)	8 (17.8%)	
Not Working/Other	162 (8.2%)	128 (7.7%)	28 (9.9%)	6 (13.3%)	
Education status					
Bachelor’s degree or higher	1031 (52.4%)	833 (50.9%)	162 (56.4%)	36 (83.7%)	
College/Technical school	645 (32.8%)	547 (33.4%)	* 92–96	* 2–6	
High school or less	292 (14.8%)	258 (15.8%)	* 29–33	* 1–5	0.0002
Charlson Comorbidity Group (2 years prior)					
No hospitalization	985 (49.3%)	808 (48.6%)	149 (51.7%)	28 (62.2%)	0.2362
0 score	914 (45.8%)	767 (46.1%)	* 131–135	* 12–16	
1	65 (3.3%)	* 60–64	* 2–6	* 1–5	
2	24 (1.2%)	* 19–23	* 1–5	* 1–5	
3+	9 (0.5%)	* 4–8	* 1–5	0 (0.0%)	
Follow-up from start to end of study period in months					
Mean (SD)	22.3 (6.7)	22.1 (6.8)	22.9 (6.3)	24.0 (7.0)	0.0443
Median (Q1–Q3)	21 (18–26)	21 (18–26)	22 (19–27)	22 (19–28)	0.0159
Reason to end of follow-up					
End of study	1974–1978	1645 (98.9%)	* 283–287	* 40–44	
New breast cancer	18	* 14–18	* 1–5	* 1–5	
Death	* 1–5	* 1–5	0 (0.0%)	0 (0.0%)	0.769

NOTE: Due to the small value of “missing data”, it has been excluded from Table 1. BI-RADS: Breast Imaging Reporting and Data System. * Small cells or ranges to prevent back calculation

**Table 2:** Breast cancer risk assessment and screening events from start of study to end of follow-up period, stratified by breast cancer risk level.

Risk Assessment and Breast Cancer Screening Events and Measures	Total *N* = 1997	Average Risk *N* = 1664	Higher than Average Risk *N* = 288	High Risk *N* = 45	*p*-Value
**Risk assessment events**					
Number of PRS test and risk assessment events					
Mean (SD)	1.00 (0.00)	1.00 (0.00)	1.00 (0.00)	1.00 (0.00)	1.00 (0.00)
Median (Q1–Q3)	1 (1–1)	1 (1–1)	1 (1–1)	1 (1–1)	NA
Min–max	1–1	1–1	1–1	1–1	
No. of participants who used the resource (%)	1997 (100%)	1664 (100%)	288 (100%)	45 (100%)	
Number of genetic counseling events					
Mean (SD)	1.00 (0.00)	1.00 (0.00)	1.00 (0.00)	1.00 (0.00)	NA
Median (Q1-Q3)	1 (1–1)	1 (1–1)	1 (1–1)	1 (1–1)	NA
Min–max	1–1	1–1	1–1	1–1	
No. of participants who used the resource (%)	211 (11%)	117 (7%)	49 (17%)	45 (100%)	
**Breast cancer screening events**					
Number of screening mammogram events					
Mean (SD)	1.18 (0.42)	1.15 (0.38)	1.32 (0.52)	1.40 (0.54)	<0.0001
Median (Q1–Q3)	1 (1–1)	1 (1–1)	1 (1–2)	1 (1–2)	<0.0001
Min–max	1–3	1–3	1–3	1–3	
No. of participants who used the resource (%)	1801 (90%)	1481 (89%)	275 (95%)	45 (100%)	
Number of diagnostic mammogram events					
Mean (SD)	1.61 (0.89)	1.58 (0.81)	1.82 (1.22)	1.43 (1.13)	0.3495
Median (Q1–Q3)	1 (1–2)	1 (1–2)	1 (1–2)	1 (1–1)	0.3943
Min–max	1–6	1–5	1–6	1–4	
No. of participants who used the resource (%)	203 (10%)	168 (10%)	28 (10%)	7 (16%)	
Number of breast MRI/ultrasound events					
Mean (SD)	1.41 (0.78)	1.40 (0.80)	1.43 (0.79)	1.44 (0.73)	0.9676
Median (Q1–Q3)	1 (1–2)	1 (1–2)	1 (1–2)	1 (1–2)	0.8701
Min–max	1–5	1–5	1–4	1–3	
No. of participants who used the resource (%)	147 (7%)	108 (6%)	23 (8%)	16 (36%)	
Number of diagnostic test (diagnostic mammogram, MRI, ultrasound, biopsy, genetics test)					
Mean (SD)	2.29 (1.74)	2.32 (1.78)	2.29 (1.69)	2.00 (1.50)	0.7547
Median (Q1–Q3)	2 (1–3)	2 (1–3)	2 (1–3)	1 (1–3)	0.5999
Min–max	1–11	1–11	1–8	1–6	
No. of participants who used the resource (%)	265 (13%)	205 (12%)	42 (15%)	18 (40%)	

PRS: Polygenic risk score, MRI: Magnetic resonance imaging.

**Table 3:** Breast cancer risk-stratified screening-related costs per person from start of study to end of follow-up period of participants who used the resource in 2022 CAD.

Breast Cancer Screening Event and Measures		Total *N* = 1997	Average Risk *N* = 1664	Higher than Average Risk *N* = 288	High Risk *N* = 45	*p*-Value
Overall costs *						
	Total cost	1,074,117	866,288	167,114	40,716	
	Mean cost (SD)	538 (181)	521 (163)	580 (192)	905 (269)	<0.0001
	Median cost (Q1–Q3)	458 (455–575)	458 (455–559)	474 (456–686)	805 (701–1056)	<0.0001
	Min–max cost	339–1742	339–1724	339–1716	701–1742	
	No. of participants who used the resource (%)	1997 (100%)	1664 (100%)	288 (100%)	45 (100%)	
Risk assessment cost **						
	Total cost	770,368	627,123	115,711	27,534	
	Mean cost (SD)	386 (77)	377 (64)	402 (93)	612 (15)	<0.0001
	Median cost (Q1–Q3)	355 (355–370)	355 (355–370)	355 (355–370)	600 (600–627)	<0.0001
	Min–max cost	339–641	339–641	339–641	600–641	
	No. of participants who used the resource (%)	1997 (100%)	1664 (100%)	288 (100%)	45 (100%)	
Total screening cost ***						
	Total cost	216,813	173,341	37,058	6414	
	Mean cost (SD)	120 (43)	117 (40)	135 (54)	143 (55)	<0.0001
	Median cost (Q1–Q3)	102 (101–104)	102 (101–104)	104 (101–205)	104 (101–204)	<0.0001
	Min–max cost	94–336	94–336	101–312	101–311	
	No. of participants who used the resource (%)	1801 (90%)	1481 (89%)	275 (95%)	45 (100%)	
Total diagnostic cost ****						
	Total cost	86,936	65,824	14,345	6768	
	Mean cost (SD)	328 (191)	321 (187)	342 (203)	376 (212)	0.4493
	Median cost (Q1–Q3)	253 (223–366)	253 (228–346)	261 (217–416)	306 (238–412)	0.3432
	Min–max cost	86–1267	86–1267	133–1033	161–898	
	No. of participants who used the resource (%)	265 (13%)	205 (12%)	42 (15%)	18 (40%)	
Screening mammogram cost						
	Total cost	134,138	107,264	22,907	3968	
	Mean cost (SD)	74 (27)	72 (25)	83 (33)	88 (34)	<0.0001
	Median cost (Q1–Q3)	63 (62–64)	63 (62–64)	64 (62–127)	64 (62–127)	0.0005
	Min–max cost	43–200	43–200	62–195	62–194	
	No. of participants who used the resource (%)	1801 (90%)	1481 (89%)	275 (95%)	45 (100%)	
Non-OBSP overhead screening cost						
	Total cost	1485	512	359	615	
	Mean cost (SD)	59 (19)	57 (17)	60 (21)	61 (22)	0.883
	Median cost (Q1–Q3)	51 (51–51)	51 (51–51)	51 (51–51)	51 (51–51)	0.8738
	Min–max cost	51–102	51–102	51–102	51–102	
	No. of participants who used the resource (%)	25 (1%)	9 (1%)	6 (2%)	10 (22%)	
Diagnostic mammogram cost						
	Total cost	22,904	18,571	3566	768	
	Mean cost (SD)	113 (72)	111 (67)	127 (93)	110 (87)	0.5142
	Median cost (Q1–Q3)	97 (65–136)	99 (65–126)	94 (65–173)	87 (65–106)	0.8862
	Min–max cost	11–440	11–440	43–388	43–301	
	No. of participants who used the resource (%)	203 (10%)	168 (10%)	28 (10%)	7 (16%)	
Overhead diagnostic cost						
	Total cost	15,776	11,934	2663	1178	
	Mean cost (SD)	60 (23)	58 (20)	63 (30)	65 (29)	0.2096
	Median cost (Q1–Q3)	51 (51–51)	51 (51–51)	51 (51–51)	51 (51–51)	0.3183
	Min–max cost	51–154	51–154	51–154	51–154	
	No. of participants who used the resource (%)	265 (13%)	205 (12%)	42 (15%)	18 (40%)	
Breast MRI/ultrasound cost						
	Total cost	11,768	7171	1645	2951	
	Mean cost (SD)	80 (82)	66 (72)	72 (66)	184 (99)	<0.0001
	Median cost (Q1–Q3)	37 (36–74)	37 (36–72)	37 (36–71)	187 (129–205)	<0.0001
	Min–max cost	35–478	35–478	35–304	36–453	
	No. of participants who used the resource (%)	147 (7%)	108 (6%)	23 (8%)	16 (36%)	
Breast biopsy cost						
	Total cost	9111	7188	1290	632	
	Mean cost (SD)	228 (151)	218 (138)	215 (153)	* NA	0.021
	Median cost (Q1–Q3)	170 (86–311)	169 (88–300)	180 (82–291)	* NA	0.2642
	Min–max cost	81–657	82–657	81–476	632–632	
	No. of participants who used the resource (%)	40 (2%)	33 (2%)	* 2–6	* 1–5	
Genetic cost						
	Total cost	1078	660	380	38	
	Mean cost (SD)	98 (57)	94 (50)	* NA	* NA	0.4335
	Median cost (Q1–Q3)	74 (39–155)	74 (39–151)	* NA	* NA	0.1982
	Min–max cost	38–171	39–155	39–171	38–38	
	No. of participants who used the resource (%)	11 (1%)	7 (0%)	* 1–5	* 1–5	
OBSP administrative cost						
	Total cost	36,494	29,469	6202	823	
	Mean cost (SD)	20 (7)	20 (7)	23 (9)	22 (9)	<0.0001
	Median cost (Q1–Q3)	18 (18–18)	18 (18–18)	18 (18–35)	18 (18–18)	<0.0001
	Min–max cost	18–53	18–53	18–53	18–53	
	No. of participants who used the resource (%)	1785 (89%)	1475 (89%)	272 (94%)	38 (84%)	
OBSP screening facility cost						
	Total cost	44,695	36,096	7591	1008	
	Mean cost (SD)	25 (9)	24 (8)	28 (11)	27 (11)	<0.0001
	Median cost (Q1–Q3)	22 (22–22)	22 (22–22)	22 (22–43)	22 (22–22)	<0.0001
	Min–max cost	16–65	16–65	16–65	16–65	
	No. of participants who used the resource (%)	1785 (89%)	1475 (89%)	272 (94%)	38 (84%)	
OBSP diagnostic follow-up cost						
	Total cost	26,300	20,300	4800	1200	
	Mean cost (SD)	116 (45)	115 (41)	123 (54)	120 (63)	0.5524
	Median cost (Q1–Q3)	100 (100–100)	100 (100–100)	100 (100–100)	100 (100–100)	0.622
	Min–max cost	100–300	100–300	100–300	100–300	
	No. of participants who used the resource (%)	226 (11%)	177 (11%)	39 (14%)	10 (22%)	

* Overall cost = Risk assessment cost + Total screening cost + Total diagnostic cost. ** Risk assessment cost = PRS test + Risk assessment letter + Genetic counseling cost. *** Total screening cost = OHIP screening mammogram + OBSP administrative + OBSP facility + Non-OBSP overhead screening cost. **** Total diagnostic cost = Diagnostic mammogram + Ultrasound + MRI + Biopsy + Diagnostic genetic + OBSP diagnostic follow-up + Overhead diagnostic cost. OBSP: Ontario Breast Screening Program, MRI: Magnetic resonance imaging.

**Table 4:** Overall healthcare utilization costs for participants who used the resource by breast cancer risk level in 2022 CAD.

Overall Healthcare Costs	Total *N* = 1997	Average Risk *N* = 1664	Higher than Average Risk *N* = 288	High Risk *N* = 45	*p*-Value
Total cost	12,285,985	10,500,857	1,552,519	232,609	
Mean cost (SD)	6152 (18,243)	6311 (19,641)	5391 (8325)	5169 (7676)	0.6848
Median cost (Q1–Q3)	2890 (1555–5523)	2920 (1560–5584)	2598 (1437–5436)	3138 (2200–4740)	0.4852
Min–max cost	355–604,893	355–604,893	568–72,552	1327–49,762	
No. of participants who used the resource (%)	1997 (100%)	1664 (100%)	288 (100%)	45 (100%)	

## Data Availability

Parts of the material underlying this article are based on data and information provided by Ontario Health (Cancer Care Ontario). Ontario Health is prohibited from making the data used in this research publicly accessible if it includes potentially identifiable personal health information and/or personal information as defined in Ontario law, specifically the Personal Health Information Protection Act and the Freedom of Information and Protection of Privacy Act. Upon request, data de-identified to a level suitable for public release may be provided.

## Appendix A

### Supplementary Methods

**Table A1:** Databases and data variables descriptions.

Databases	Data Variables
Demographics/Population	
Local Health Integration Network (LHIN)	14 LHINs, operating as Home and Community Care Support Services across Ontario, providing home care and long-term care home placement services and facilitation of access to community services
Postal Code Conversion File (PCCF)	Allows for the matching of six-digit postal codes to standard census geographies.
Registered Persons Database (RPDB)	Basic population and demographic information (age, sex, location of residence, date of birth and date of death for deceased individuals)
Health services	
CIHI (Canadian Institute for Health Information) Continuing Care Reporting System (CCRS)	Complex and continuing care
CIHI Discharge Abstract Database (DAD)	Inpatient hospitalizations
CIHI Same Day Surgery (SDS)	All day surgical procedures
Ontario Home Care Database (HCD)	Home care visits
CIHI National Ambulatory Care Reporting System (NACRS)	Emergency department visits, outpatient clinic visits, same day surgery
CIHI National Rehabilitation Reporting System (NRS)	Inpatient rehabilitation
Ontario Breast Screening Program (OBSP)	Screening program encouraging people in Ontario to get screened for breast cancer via two groups of people eligible for breast cancer screening in Ontario (average risk and high risk)
Ontario Drug Benefit (ODB) Formulary	Drug prescriptions
Ontario Health Insurance Plan (OHIP) billings	Physician visits, non-physician (allied health) visits, laboratory services
Ontario Mental Health Reporting System (OMHRS)	Analyses and reports on information submitted to CIHI about all individuals receiving adult mental health services in Ontario
Controlled Use Datasets	
New Drug Funding Program (NDFP)	Chemotherapies (IV)
Ontario Cancer Registry (OCR)	Cancer diagnosis
Other	
Ontario Case Costing Initiative (OCCI)	Costs of acute inpatient, day surgery, ambulatory care cases, mental health, rehabilitation and complex continuing care
